# Electrically Tunable VO_2_-Based Metasurface for Shared-Aperture Terahertz Molecular Fingerprint Sensing and Programmable Wavefront Manipulation

**DOI:** 10.3390/nano16181170

**Published:** 2026-09-16

**Authors:** Jiahe Wang, Wenlong Li, Xilai Zhao, Wenye Ji, Xiongyu Liang, Tong Cai, Zhenxu Wang, Jiangang Liang

**Affiliations:** Air and Missile Defense College, Air Force Engineering University, Xi’an 710051, China; wangjiahe2025@163.com (J.W.); wenll011204@163.com (W.L.); zxl511178738@126.com (X.Z.); jiwenyewave@163.com (W.J.); xiongyu_liang@alumni.hust.edu.cn (X.L.); caitong326@sina.cn (T.C.)

**Keywords:** terahertz metasurface, over-coupled regime, vanadium dioxide, molecular fingerprint sensing, wavefront manipulation, refractive index sensing

## Abstract

Metasurfaces enable spatially programmable control of far-field wavefronts, while broadband molecular sensing relies on strong near-field confinement and enhanced light–matter interaction. The mismatch between far-field radiation and near-field localization makes their monolithic integration in a single shared aperture challenging. Here, we report an electrically tunable terahertz (THz) VO_2_-based metasurface designed under temporal coupled mode theory (TCMT). The device adopts a metal–insulator–metal (MIM) architecture integrating a VO_2_ microbridge and bowtie antenna, enabling bias-controlled switching between molecular fingerprint sensing and programmable wavefront manipulation in a shared aperture. At zero bias, the metasurface operates in an over-coupled regime, supporting a broadband low Q resonance over 0.8–1.2 THz with strong local field enhancement for molecular fingerprint sensing demonstration, serving as a passive near-field sensing mode. Under the metallic state condition induced by the VO_2_ phase transition, the unit cell exhibits an approximately 180° reflection phase shift with nearly invariant amplitude. An electrically assisted programmable addressing implementation is proposed by exploiting the electrothermal switching capability of VO_2_. Each unit cell thus functions as a 1-bit programmable meta-atom. By reconfiguring the spatial coding sequence, the device realizes anomalous reflection and beam splitting, which are further extended to refractive index sensing: thin analyte layers (<10 μm) are detected via anomalous reflection, whereas thicker layers are detected via beam splitting when the anomalous beam is suppressed. This constitutes a far-field active sensing mode. Both functionalities originate from the same over-coupled radiative loss mechanism: enhanced near fields enable fingerprint sensing, while the open radiative channel supports far-field refractive index sensing through programmed phase coding. This shared aperture design unifies near-field and far-field, passive and active sensing, offering a compact platform for THz integrated sensing and communication.

## 1. Introduction

Terahertz band (0.1–10 THz) combines large communication bandwidth, nonionizing detection, and access to molecular fingerprint spectra [1,2,3,4], making it attractive for next generation high speed wireless links, label free biosensing, and intelligent sensing systems. These applications place competing demands on electromagnetic control [4,5,6,7,8]. Efficient wavefront manipulation requires strong radiative coupling to free space [9,10], whereas sensitive acquisition of intrinsic molecular information requires electromagnetic energy to be confined near the device surface to enhance light–matter interaction [11]. This tradeoff has generally led to discrete architecture in which resonant sensing elements and beam forming antenna arrays are designed separately [12,13,14,15]. In addition, many THz fingerprint sensors rely on multiple narrowband resonators to cover several molecular absorption features [16,17,18], which increases system complexity while limiting modulation efficiency. A multifunctional THz platform that combines broadband molecular fingerprint sensing with dynamic wavefront manipulation in a shared aperture is therefore highly desirable for future integrated sensing and communication [19,20,21,22]. Moreover, programmable wavefront control provides a promising pathway toward far-field refractive index sensing, which can complement near-field molecular fingerprint sensing within the same aperture.

A route to reconciling these requirements is to engineer the exchange of energy between the metasurface and free space [23,24,25]. Temporal coupled mode theory provides a unified description of this exchange, while metasurfaces offer a flexible platform for combining both near-field confinement and spatial phase. For a metasurface backed by a metallic ground plane, the electromagnetic response can be described as a single port reflective resonator whose spectrum is governed by the competition between radiative loss rates ($\gamma_{r}$) and non-radiative loss rates ($\gamma_{nr}$) [26]. Critical coupling occurs when the two loss channels are balanced ($\gamma_{r}=\gamma_{nr}$) [27,28,29], yielding near perfect absorption and a narrowband, high Q resonance. Such resonances are useful for narrowband absorption enhancement, but they are less suitable for broadband molecular fingerprint sensing, where absorption features are distributed over a wider spectral window. In the over-coupled regime, radiative loss dominates ($\gamma_{r}>\gamma_{nr}$), the resonance broadens and the Q factor decreases, yet strong local field enhancement can be maintained over a broad bandwidth [30,31,32]. The same enhanced radiative channel also improves coupling to free space, providing the physical basis for wavefront manipulation and far-field refractive index sensing within the same aperture. The molecular sensing results are demonstrated through electromagnetic enhancement of analyte-induced THz responses rather than quantitative concentration determination.

The dual functionality of the proposed metasurface therefore originates from the same coupling regime engineering rather than from the simple combination of two independent modules. In the over-coupled regime, the resonator remains strongly coupled to free space while maintaining enhanced local fields near the bowtie gap. The enhanced surface field provides the light–matter interaction required for molecular fingerprint sensing, whereas the open radiative channel enables the reflected wave to be redistributed in the far-field once a spatial phase gradient is electrically programmed. Broadband sensing and wavefront manipulation are therefore physically linked by radiative loss modulation in the same over-coupled MIM resonator [33,34,35]. Moreover, by programming the coding sequence, the far-field beam response can be further exploited for refractive index sensing, thereby extending the unified platform to include both near-field molecular fingerprint sensing and far-field programmable refractive-index sensing. Molecular fingerprint sensing and beam manipulation represent two fundamental electromagnetic capabilities required by future sensing communication front ends. Wavefront manipulation follows the Generalized Snell’s Law: when a phase gradient is imposed at an interface, the reflected wave acquires an additional transverse wave vector, leading to anomalous reflection, beam splitting, and related effects. To achieve electrically reconfigurable phase control, we use vanadium dioxide (VO_2_) as a tunable phase change material. VO_2_ undergoes an insulator to metal transition near 68 °C, accompanied by a conductivity change of three to four orders of magnitude [36]. By triggering this transition through electrothermal actuation and by engineering the unit cell geometry [37,38], the metasurface can produce an approximately 180° phase contrast between two states while maintaining nearly matched reflection amplitudes. The phase response can therefore be encoded as a 1-bit state, with “0” and “1” corresponding to 0° and 180°, respectively. Dynamic programming of the spatial coding sequence enables anomalous reflection, beam scanning, and multi beam splitting without mechanical movement or structural reconfiguration.

Although over-coupled resonant metasurfaces have been widely explored for broadband molecular sensing, and VO_2_-based phase change metasurfaces and electrically programmable THz metasurfaces have enabled dynamic wavefront manipulation, these functionalities are generally optimized independently or integrated through spatially separated functional elements. In contrast, the proposed metasurface not only realizes both sensing and beamforming functions through the same over-coupled MIM resonant mode, but also extends the programmable wavefront control to far-field refractive index sensing, thereby unifying near-field passive molecular fingerprint sensing and far-field active refractive index sensing within one shared aperture. By engineering the balance between radiative and non-radiative losses, the shared aperture structure simultaneously provides enhanced near-field confinement for molecular fingerprint sensing and an efficient radiative channel for programmable wavefront control. This approach establishes a physically unified platform where two distinct electromagnetic functionalities originate from the same resonant mechanism. It should be noted that the molecular fingerprint sensing demonstrated in this work focuses on enhanced electromagnetic interaction between the metasurface resonance and analyte absorption features. Quantitative biochemical parameters, such as analyte concentration and detection limit, require additional experimental calibration and are beyond the scope of the present electromagnetic design study.

Here, we develop an electrically tunable VO_2_-based THz metasurface that integrates molecular sensing and communication-oriented wavefront control functions at the device level. The metasurface adopts an MIM configuration in which a VO_2_ microbridge is incorporated into a bowtie antenna. Without external bias, the device supports a low Q over-coupled resonance across 0.8–1.2 THz, producing enhanced local fields for high sensitivity THz molecular fingerprint sensing. Under electrical bias, selected VO_2_ microbridges undergo a phase transition, and the same radiatively open resonant aperture is converted into a programmable reflective array with a spatial phase gradient. At 1 THz, the unit cell retains a nearly unchanged reflection amplitude before and after the phase transition while providing a 180° phase difference, thereby forming a 1-bit programmable element. By electrically assisted programmable addressing of selected columns, the device realizes retroreflection, anomalous reflection, and beam splitting in the same physical aperture under different voltage coding sequences. In particular, the anomalous reflection and beam splitting modes are employed for refractive index sensing: thin analyte layers are more effectively detected via anomalous reflection, whereas thicker layers are better identified via beam splitting when the anomalous beam is suppressed. Importantly, the sensing and wavefront control functions share the physical structure and electrical control network. Switching between the electromagnetic functionalities can be achieved by controlling the VO_2_ phase state through electrical addressing. The practical switching dynamics depend on the electrothermal response of the device. These results show that strong field confinement and efficient radiation can be unified through over-coupled resonances, enabling broadband fingerprint sensing and programmable wavefront manipulation in a single THz metasurface. This shared aperture design therefore realizes a unified integration of near and far-field, passive and active function modalities within a single THz metasurface. The strategy offers a compact route toward multifunctional THz front ends for future integrated sensing and communication systems. In this work, molecular fingerprint sensing refers to the near-field broadband sensing mode based on enhanced light–matter interaction. The electrically programmable refractive-index sensing mode is referred to as active sensing, because the sensing function relies on electrically programmable phase coding.

## 2. Results and Discussion

**Figure 1:**
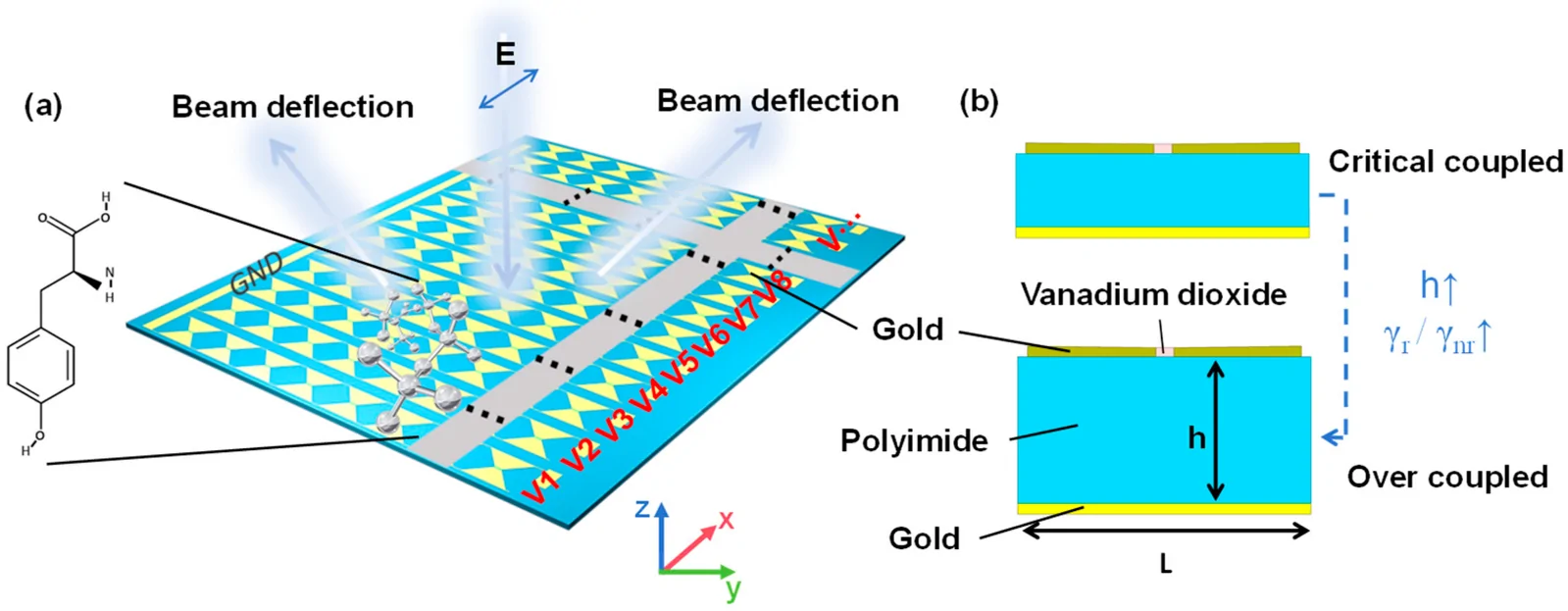
Schematic design and operating principle of the electrically tunable VO_2_-based THz metasurface. (**a**) Schematic illustration of the metasurface array integrating molecular fingerprint sensing and programmable wavefront manipulation within a shared physical aperture. The same bowtie/VO_2_/MIM resonant unit provides near-field enhancement in the unbiased sensing state and 1-bit reflective phase coding in the biased wavefront control state. The unit cell consists of a gold bowtie antenna bridged by a VO_2_ microbridge on a polyimide spacer and a gold ground plane. Electrical addressing of selected subarrays is proposed as a possible implementation strategy for reconfigurable phase coding and beam deflection. (**b**) Cross sectional view of the metal–insulator–metal unit cell. By increasing the polyimide spacer thickness, the ratio between radiative and non-radiative loss rates is tuned, allowing the metasurface to transition from the critical coupling regime to the over-coupled regime.

The designed metasurface unit cell adopts MIM architecture, as illustrated in Figure 1a. From bottom to top, the structure consists of a 200 nm thick gold ground plane in a unit cell with period L = 160 μm, a spin-coated polyimide (PI) dielectric spacer with thickness h = 15 μm, and a top gold bowtie antenna bridged by a VO_2_ microbridge. The gold ground plane suppresses transmission and forms a single port reflective resonator. The bowtie antenna strongly localizes the electric field in the gap region and enables excitation of the over-coupled mode; as shown in Figure 1b, varying the PI thickness allows the system to be tuned between critical and over-coupled regimes. The VO_2_ microbridge is positioned in the field enhanced region, where it tunes the resonance and acts as an electrothermally driven phase change element. At the array level, the metasurface is configured as a linear array of 60 addressable subcolumns, each containing 100 rows of unit cells. One end of each subarray is connected to a common ground electrode, and the other end is connected to an external voltage source, enabling dynamic and reconfigurable phase control in the THz regime. Thus, the same bowtie/VO_2_/MIM element operates as a surface field enhancing sensing unit in the unbiased state and as a 1-bit reflective phase coding unit under electrical bias. Thus, under different electrical biases, the structure can realize fingerprint spectrum sensing and refractive index sensing respectively.

The conductivity of VO_2_ is governed by temperature. At room temperature, a bandgap of approximately 0.7 eV between bonding and antibonding states near the Fermi level gives VO_2_ an insulating response. When the temperature exceeds the phase transition threshold, electronic correlations weaken and a broad, partially filled conduction band crosses the Fermi level, allowing carriers to move more freely and producing metallic behavior. In a potential experimental realization, an electrothermally assisted switching strategy could be employed. The metasurface array may be uniformly heated to approximately 60 °C to bring VO_2_ close to its insulator to metal transition threshold while remaining in the insulating state. An external bias voltage would then be selectively applied to individual subarrays. The resulting current flow through the VO_2_ microbridge and the adjacent metallic electrodes would generate localized Joule heating. Given the preheated initial condition, only a modest additional temperature increase would be required to drive the targeted columns to approximately 68 °C, thereby triggering the insulator to metal phase transition of VO_2_. Similar approaches have been reported [39] in electrically addressable VO_2_-based terahertz metasurfaces, where a preheated substrate temperature close to the transition temperature was combined with localized Joule heating to achieve programmable switching. In those systems [39], introducing highly thermally conductive layers was employed to reduce thermal cross-talk between neighboring addressable regions. This preheating assisted scheme would reduce the required driving power per column and could also mitigate thermal cross-talk between neighboring channels, enabling spatially selective phase encoding.

The fingerprint sensing mode does not require preheating and can operate at room temperature. However, this does not mean that it must operate only at room temperature. When the device is preheated to 60 °C, VO_2_ remains in the insulating state and the system remains in the over-coupled regime; therefore, fingerprint sensing also works properly at this temperature. In this way, fingerprint sensing and refractive-index sensing can share the same 60 °C preheating platform, and switching between them is achieved solely by electrical biasing without changing the global temperature. The corresponding resonance spectrum and molecular detection results at 60 °C are provided in Figure S4. The thermal actuation required for the VO_2_ phase transition may affect the integrity of thermally sensitive biological analytes. Therefore, the electrically programmable wavefront sensing mode is more suitable for thermally stable analytes, or can be implemented in a time-sequential manner, in which molecular fingerprint acquisition is completed before thermal actuation. The preheating-assisted phase transition scheme is primarily used to reduce the driving power. In practical biosensing, some analytes remain stable on a heated substrate, while others do not. Thus, the applicability of this mode to an analyte depends on its thermal stability.

The metasurface has two operating states, both of which can be understood within the TCMT framework of radiative loss modulation. In the absence of an external bias, VO_2_ remains insulating and the metasurface behaves as a uniform radiating aperture. The resonance spans 0.8–1.2 THz, and the surface electric field is enhanced throughout this band, making this state suitable for broadband molecular fingerprint sensing demonstration. The enhanced field is concentrated near the bowtie gap and the VO_2_ microbridge, so analyte molecules deposited on the top surface overlap efficiently with the field enhanced region and experience strong light–matter interaction. When selected VO_2_ elements are switched to the metallic state, the corresponding columns exhibit a phase change and change the unit cell reflection phase by approximately 180°. The array radiation is no longer spatially uniform. By programming the “0”/“1” coding sequence across the columns, a spatial phase gradient is imposed on the metasurface, and the incident THz wave is redirected according to the prescribed sequence. The far-field beam response can thus be exploited for refractive index sensing, since analyte induced phase perturbations modulate the anomalous reflection and beam splitting efficiency. The device thus switches from the near-field passive sensing mode to the far-field active sensing mode without changing the physical aperture.

### 2.1. Coupled Mode Theory Analysis and Molecular Fingerprint Sensing

Because the gold ground plane is much thicker than the skin depth in the operating band, transmission through the metasurface is negligible. The structure can therefore be treated as a single port resonator. In the absence of transmitted waves, the coupled-mode model of absorption for a resonator as a function of frequency ω explains this behavior.

(1) $$A=\frac{4\gamma_{r}\gamma_{nr}}{{\left(\omega -\omega_{r}\right)}^{2}+{\left(\gamma_{r}+\gamma_{nr}\right)}^{2}}$$

where $\gamma_{r}$ and $\gamma_{nr}$ denote the radiative and non-radiative loss rates, respectively, and $\omega_{0}$ is the resonant angular frequency. At resonance, this expression reduces to

(2) $$A_{0}=\frac{4\gamma_{r}\gamma_{nr}}{{\left(\gamma_{r}+\gamma_{nr}\right)}^{2}}=\frac{4\gamma_{r}/\gamma_{nr}}{{\left(1+\gamma_{r}/\gamma_{nr}\right)}^{2}}$$

This expression shows that absorption is determined by the relative magnitudes of the radiative and non-radiative loss channels. In our structure, the PI spacer thickness provides an effective handle for tuning $\gamma_{r}$, whereas $\gamma_{nr}$—mainly associated with metallic ohmic loss and dielectric absorption—changes only weakly.

**Figure 2:**
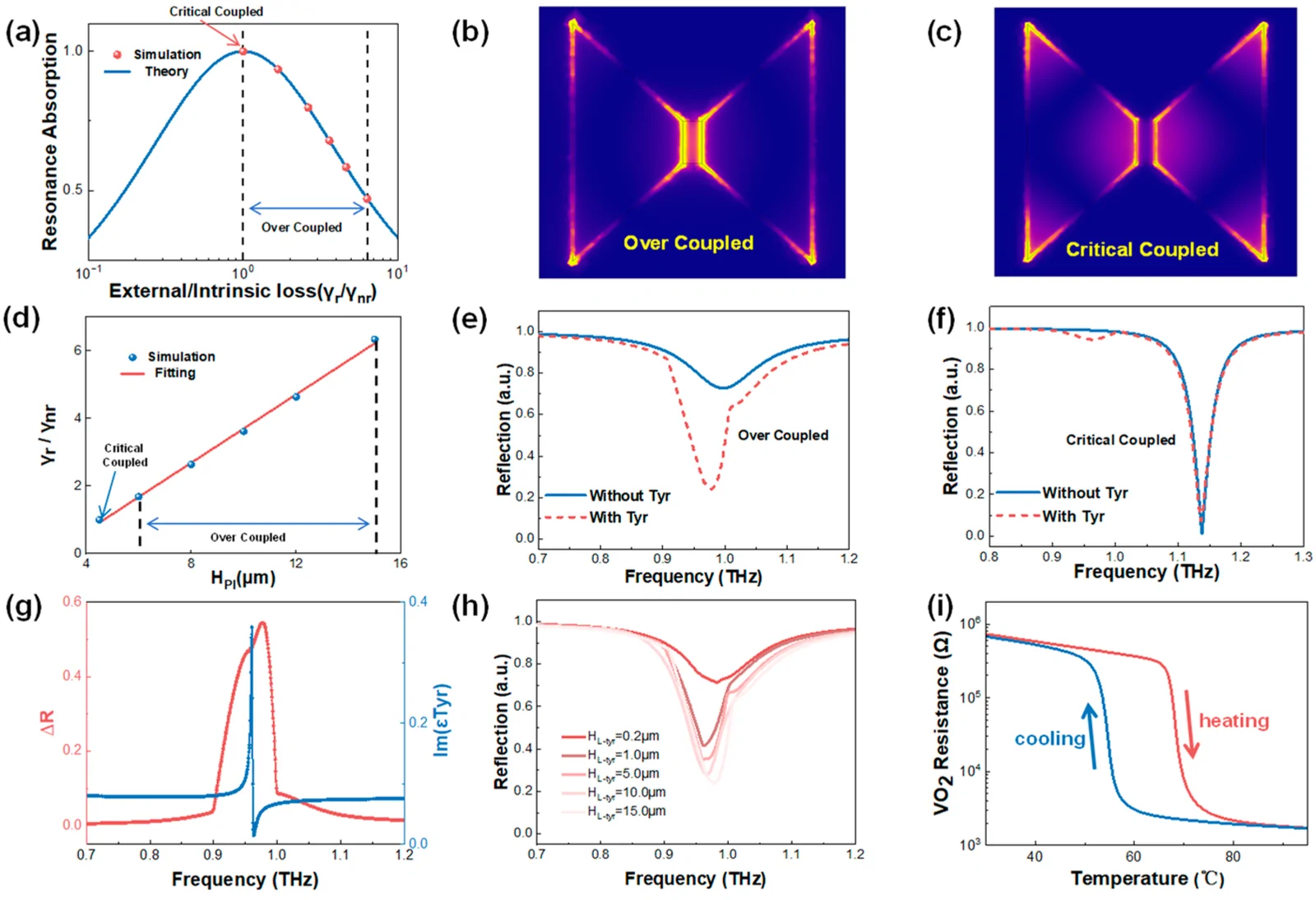
Coupled mode analysis and broadband molecular fingerprint sensing performance. (**a**) Temporal coupled mode theory analysis showing the dependence of absorption on the ratio between radiative and non-radiative loss rates, highlighting the transition from critical coupling to over coupling. (**b**,**c**) Simulated electric field distributions of the metasurface in different coupling regimes, showing stronger field localization in the over-coupled structure. (**d**) Dependence of the radiative-to-non-radiative loss ratio $\gamma_{r}/\gamma_{nr}$ on the PI spacer thickness. The simulated data reveals the transition from critical coupling to over coupling with increasing PI thickness. (**e**,**f**) Reflection spectra of the metasurface with and without L-tyrosine under over-coupled and critically coupled conditions, respectively. (**g**) Reflectance difference spectrum of the over-coupled metasurface compared with the imaginary part of the permittivity of L-tyrosine, confirming the sensing of the molecular fingerprint absorption near 0.96 THz. (**h**) Simulated reflection spectra of the metasurface with different L-tyrosine thicknesses. (**i**) Experimentally measured temperature-dependent resistance of the VO_2_ film.

Figure 2a shows TCMT fits to the reflection spectra for PI thicknesses of 4.5 μm and 15 μm, illustrating the transition between the critical coupling and over-coupled regimes. When $\gamma_{r}=\gamma_{nr}$, the incident energy is dissipated by internal loss in the resonator, the absorption approaches unity, and perfect absorption is obtained; this is the critical coupling regime. When $\gamma_{r}>\gamma_{nr}$, the system enters the over-coupled regime. In this case, the radiative channel dominates, the reflection minimum no longer reaches zero, and the resonance linewidth broadens. The electric field in the VO_2_ gap region remains strongly enhanced, which promotes surface light–matter interaction and provides favorable conditions for molecular fingerprint sensing. At the same time, the open radiative channel allows each unit cell to direct electromagnetic energy into the far-field once spatial phase encoding is introduced, rather than trapping the energy within the metasurface.

The extracted parameters in Table 1 quantitatively confirm the transition between coupling regimes. When the PI thickness is 4.5 μm, the radiative and non-radiative loss rates are approximately equal ($\gamma_{r}$/$\gamma_{nr}$ ≈ 1), and the system operates in the critical coupling regime. When the PI thickness is increased, the radiative loss increases significantly, and the system enters the over-coupled regime ($\gamma_{r}/\gamma_{nr}$ > 1). Since the metallic structure remains unchanged, the non-radiative loss rate remains nearly constant, confirming that the coupling regime is primarily controlled by the PI thickness through modulation of the radiative loss channel. Using the extracted parameters in Table 1, the TCMT fitting results show R^2^ > 0.99, as presented in Figure S1, verifying the reliability of the extracted loss rates.

This radiatively open but field-enhanced state is the physical connection between the two functions demonstrated here. For sensing, the accessible near-field increases the overlap between the molecular layer and the resonant electromagnetic mode, thereby amplifying fingerprint absorption. For wavefront manipulation, the same dominant radiative channel ensures that the phase coded unit cells contribute efficiently to coherent far-field interference. Therefore, molecular fingerprint sensing and programmable beam manipulation are not two unrelated effects implemented on different devices; they are two bias selected uses of the same over-coupled resonant aperture.

The coupling regime of the present metasurface is controlled by the PI spacer thickness. For h = 4.5 μm, the radiative and non-radiative loss rates are approximately balanced near 1.1 THz, and the system approaches critical coupling. The reflection dip is deep, the linewidth is narrow, and the Q factor is high, which is advantageous for narrowband absorption enhancement but unfavorable for broadband fingerprint sensing across multiple molecular resonances. Because the radiative and non-radiative channels are nearly matched, this regime is also less suitable for wavefront manipulation. Increasing the PI thickness to 15 μm enhances the radiative channel and drives the system from critical coupling into the over-coupled regime. The resonance evolves from a narrowband absorption feature into a broadband response covering 0.8–1.2 THz. As shown in Figure 2b,c, the electric field distribution changes markedly: compared with the critically coupled structure, the over-coupled structure maintains a strong localized field in the accessible gap region while broadening the resonance bandwidth and preserving efficient radiation coupling. Quantitatively, the maximum electric-field intensity in the over-coupled configuration is enhanced by approximately 2.19 times compared with the critical-coupling case. This field localized yet radiatively open regime is therefore beneficial for both molecular fingerprint sensing and beam manipulation.

Conventional THz fingerprint metasurfaces often use cascaded arrays of narrowband resonators or phase change components to cover several molecular absorption peaks. Such approaches require multiple measurements and complex post processing, which increases the detection time and system complexity. In contrast, the proposed over-coupled metasurface covers the 0.8–1.2 THz fingerprint window in a single measurement on one device, eliminating the need for multi resonator concatenation and improving spectral acquisition efficiency. In the simulation setup, unit cell boundary conditions were applied in the x- and y-directions, and open boundary conditions were applied in the z direction. A Floquet port was used to excite a TM-polarized plane wave from the top. The L-tyrosine layer was modeled as a homogeneous thin film, with its dielectric function described by parameters extracted from the experimentally measured complex permittivity reported in [40]. Figure 2d shows the variation of $\gamma_{r}/\gamma_{nr}$ with PI spacer thickness, increasing from 1.02 to 6.34, which quantitatively evidences the critical-to-over-coupled transition. The fitting yields a coefficient of determination R^2^ > 0.99, confirming the robustness of the parameter extraction. Figure 2e,f compares the reflection spectra [41] of the over-coupled and critically coupled regimes with and without L-tyrosine. The L-tyrosine example serves as a representative molecular fingerprint sensing to verify the enhanced light–matter interaction enabled by the over-coupled resonance. In the over-coupled regime, the broadband, low Q resonance clearly reveals the L-tyrosine fingerprint absorption near 0.96 THz. In the critically coupled regime, only a narrow resonance is excited, and the molecular fingerprint feature is barely discernible. Figure 2g plots the reflectance difference in the over-coupled metasurface together with the imaginary part of the permittivity of L-tyrosine, showing that the molecular absorption peak can be identified directly without additional smoothing or background correction. For two or more closely spaced fingerprint peaks within the same spectral band, other molecular fingerprint features located in the operating range of the proposed device (0.8–1.2 THz) can also be enhanced and observed through their corresponding spectral responses. The detection of multiple fingerprint features is demonstrated in Figure S2. For absorption peaks with extremely small frequency separations, additional strategies such as multi-resonant designs or spectral reconstruction methods may be required to further improve peak discrimination.

To evaluate the influence of analyte loading, the dependence of the fingerprint response on the L-tyrosine thickness was investigated. As shown in Figure 2h, the response gradually increases with increasing analyte thickness, indicating enhanced light–matter interaction enabled by the localized electromagnetic field. This thickness-dependent analysis provides insight into the sensing mechanism, but quantitative concentration sensitivity and detection limits require additional experimental calibration. Since the simulations are performed under ideal, noise-free conditions, the minimum detectable variation in analyte properties obtained numerically should not be directly interpreted as an experimental detection limit.

The temperature-dependent electrical response of VO_2_ was experimentally characterized, as shown in Figure 2i. The resistance remains nearly constant below 68 °C, indicating a stable insulating state at room temperature. When the temperature approaches 68 °C, the resistance decreases by approximately three orders of magnitude, confirming the thermally induced insulator-to-metal transition. This result verifies the feasibility of temperature-assisted VO_2_ switching for reconfigurable wavefront manipulation.

It should be noted that over-coupled resonant structures have been previously demonstrated for broadband molecular sensing. Therefore, the main contribution of this work is not to further optimize a single sensing metric, such as sensitivity or detection limit, but to extend the functionality of the over-coupled regime toward shared aperture multifunctional THz metasurfaces. The L-tyrosine layer is used as a representative molecular fingerprint analyte to verify the enhanced light–matter interaction enabled by the over-coupled resonance. The obtained spectral response demonstrates the capability of the metasurface to enhance molecular absorption signatures. In the proposed structure, the enhanced near field in the over-coupled state enables molecular fingerprint sensing, while the open radiative channel provides the basis for electrically programmable wavefront manipulation and refractive index sensing. Thus, the over-coupled resonance serves as a common electromagnetic platform connecting near-field sensing and far-field wavefront control.

In the unbiased state, the metasurface can be modeled as an LC resonant circuit [42]: the bowtie antenna gap provides the equivalent capacitance, while the metal arms act as the equivalent inductance. Under TM polarized incidence, the electric field is oriented along the bowtie opening, driving a strong resonance and pronounced field enhancement. Under TE polarized incidence, charge oscillations are not efficiently excited, and the metasurface maintains high reflectivity without an appreciable resonance. This polarization selectivity provides intrinsic THz polarization filtering, suppressing unwanted background signals and improving the signal-to-noise ratio for fingerprint sensing.

### 2.2. Beam Manipulation and Refractive Index Sensing

**Figure 3:**
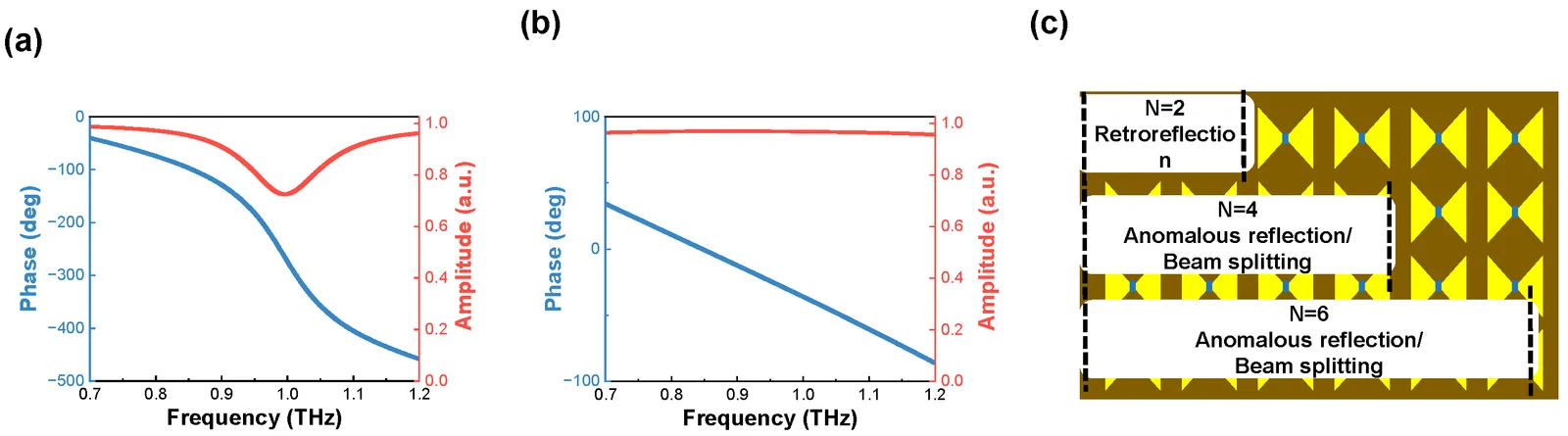
Electrically programmable 1-bit phase coding unit and coding sequences. (**a**,**b**) Simulated reflection phase and amplitude responses of the unit cell before and after the VO_2_ phase transition. The insulating and metallic states of VO_2_ provide two reflection states with an approximately 180° phase difference while maintaining nearly matched reflection amplitudes, enabling 1-bit phase coding. (**c**) Representative coding sequences for different wavefront manipulation functions. The coding periods of *N* = 2, *N* = 4, and *N* = 6 are used to realize retroreflection, anomalous reflection, and beam splitting.

The dominant radiative channel in the over-coupled regime ensures efficient energy coupling between each unit cell and free space, which is the prerequisite for array-level wavefront manipulation. Near 1 THz, the unit cell exhibits two reflection states. When VO_2_ is insulating, the unit cell corresponds to the code “1” and the reflection phase is defined as 180° (Figure 3a). When VO_2_ is switched to the metallic state by electrothermal actuation, the unit cell corresponds to the code “0” and the reflection phase changes to 0° (Figure 3b). The two states have nearly matched reflection amplitudes and an approximately π phase contrast, satisfying the key requirements for a 1-bit coding metasurface: amplitude uniformity and phase inversion.

When coding biases are applied to the subarrays, a discrete 1-bit phase distribution is formed across the metasurface. For a reflective metasurface with a spatial phase profile *φ*(*x*) along the x direction, the fields radiated by the unit cells interfere coherently in the far field, and the beam direction follows the generalized Snell law of reflection [43,44,45,46]:

(3) $$n_{i}k_{0}\sin\theta_{r}-n_{i}k_{0}\sin\theta_{i}=\frac{d\varphi }{dx}$$

where *θ_i_* is the incident angle, *θ_r_* is the reflection angle, *n_i_* is the refractive index of the surrounding medium, and *dφ*/*dx* is the phase gradient. Because each subarray can be electrically assisted with programmable addressing, the phase gradient can be programmed flexibly to realize multiple wavefront functions in the same aperture. As shown in Figure 3c, we use supercell encodings with different numbers of unit cells and different incidence angles to validate the reconfigurable wavefront control capability.

To quantitatively evaluate the wavefront manipulation performance, the desired-beam conversion efficiency was calculated from the far-field radiation pattern. We integrate the simulated signal to obtain the total input energy of the incident wave, denoted as *P_tot_*. Integrating the anomalous deflection signal yields the energy *P_ano_*. Accordingly, the efficiency of the beam deflector is defined as follows:

(4) $$\eta =\frac{P_{ano}}{P_{tot}}\times 100\%$$

For beam splitting, efficiency can be defined as follows:

(5) $$\eta =\frac{P_{+}+P_{-}}{P_{tot}}\times 100\%$$

where P_+_ and P_−_ denote the energies of the two beams in beam splitting, respectively. The reported efficiency represents the power converted into the designed radiation channels, while the remaining power is distributed among undesired diffraction orders and dissipative losses. The diffraction efficiency is limited by the binary phase quantization and metallic dissipation inherent to the VO_2_-based MIM structure. Nevertheless, the proposed design provides electrically reconfigurable functionality within a shared aperture. The corresponding coding sequences, functions and calculated desired efficiencies are summarized in Table 2.

**Figure 4:**
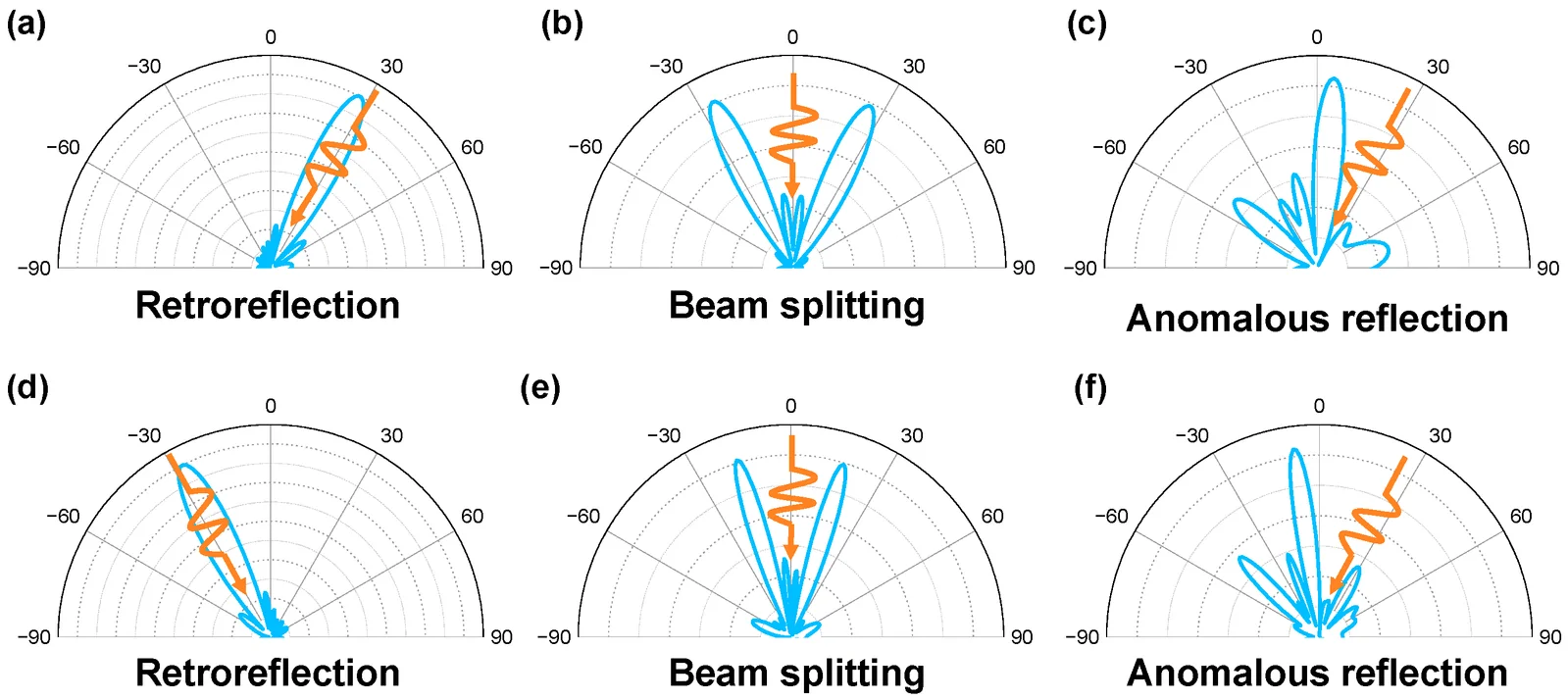
Far-field scattering patterns of the programmable THz metasurface. Simulated far-field scattering patterns under different 1-bit coding sequences and incidence conditions. (**a**,**d**) For *N* = 2, the metasurface realizes retroreflection under oblique incidence. (**b**,**e**) For *N* = 4 and *N* = 6 under normal incidence, beam splitting is achieved with dual reflected beams. (**c**,**f**) Under oblique incidence, the same coding sequences generate anomalous reflection with well-defined beam steering angles.

Figure 4a–f shows the far-field scattering patterns for the programmed functions. For *N* = 2, retroreflection is obtained (Figure 4a,d): under ±28° incidence, the reflected main lobes point toward ±28° in the reverse direction of incidence, while the specular reflection channel is suppressed by more than 14 dB. For *N* = 4 and *N* = 6 under normal incidence, beam splitting is achieved (Figure 4b,e), producing dual beams at ±26° and ±17°, respectively, with a power difference below 0.3 dB. For the same *N* = 4 and *N* = 6 under oblique incidence, anomalous reflection is excited (Figure 4c,f), and the deviation between the simulated main lobe direction and the theoretical prediction remains within 0.5°. Switching among retroreflection, beam splitting, and anomalous reflection is achieved entirely through electrical programming of the subarray voltage sources, without mechanical motion or structural reconfiguration.

The programmable beam manipulation demonstrated above can be further exploited for far-field refractive index sensing. When analyte molecules are deposited on the metasurface, the unit cell phase is perturbed, degrading the coding quality and thereby altering the far-field beam response. As the analyte thickness increases, the anomalous reflection efficiency decreases continuously. At a thickness of approximately 10 μm, the anomalous reflection beam is strongly suppressed, and reliable sensing signals can no longer be extracted using the anomalous reflection mode. Therefore, 10 μm is defined as the boundary between thin and thick layers. So, analyte layers with thicknesses below 10 μm (*λ*/30) are defined as thin layers, whereas analyte layers with thicknesses of 10 μm or greater are defined as thick layers. This classification is based on the simulated evolution of the far-field response with analyte thickness. We found that when the analyte thickness exceeds 10 μm, the anomalous reflection beam would be suppressed. For thin analyte layers, the anomalous reflection mode is employed. Taking the supercell encodings with *N* = 2 and *N* = 4 as examples, the ratio between the specular reflection energy (|*E_spe_*|^2^) and the anomalous reflection energy (|*E_ano_*|^2^) is defined as the sensing indicator. The refractive index sensitivity was quantified by calculating the variation in the sensing metric per unit refractive index change as follows:

(6) $$S=\frac{{\left|E_{spe}\right|}^{2}/{\left|E_{ano}\right|}^{2}}{\Delta n}$$

**Figure 5:**
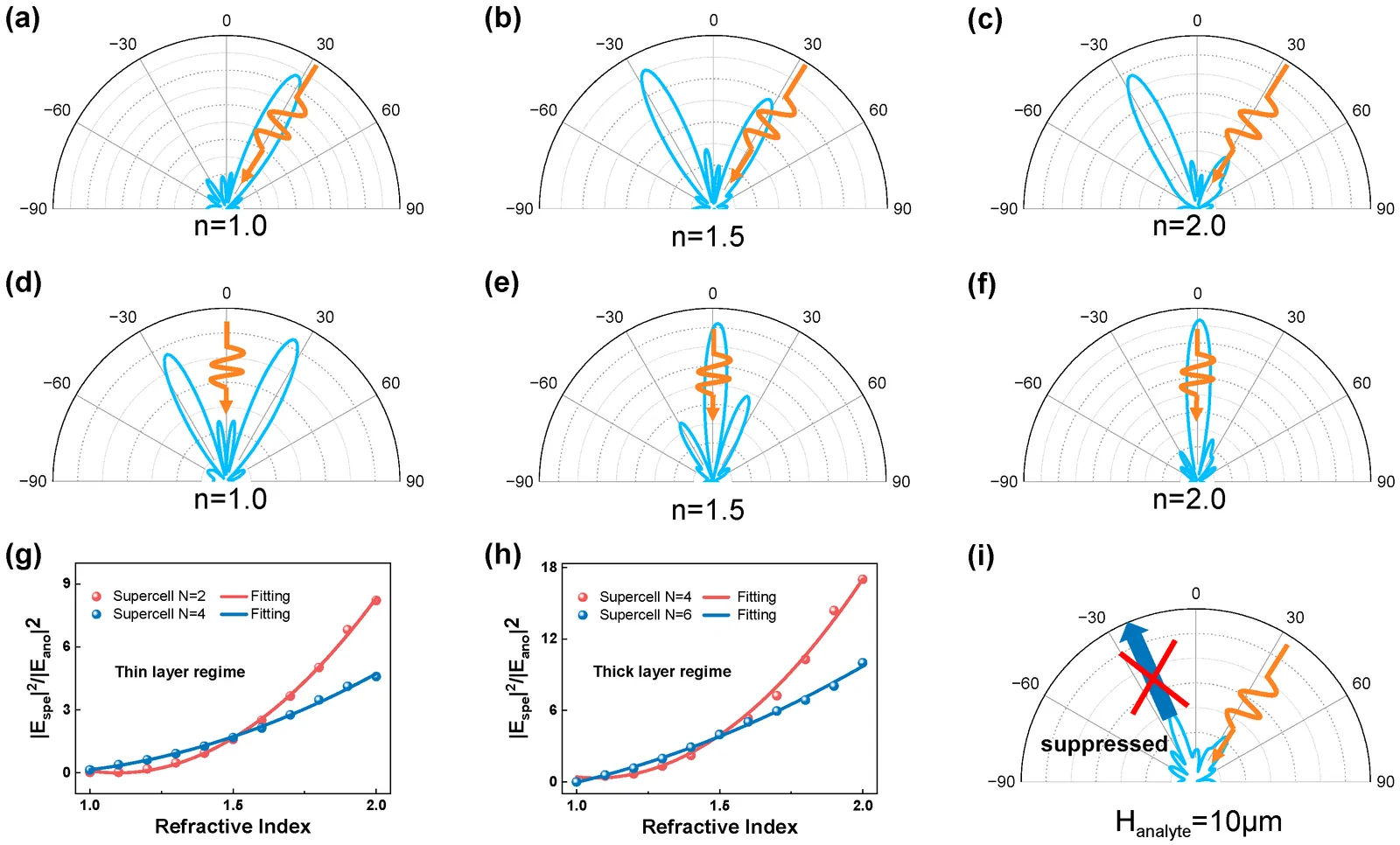
Refractive index sensing based on programmable beam manipulation. (**a**–**c**) Far-field scattering patterns for *N* = 2 at 27.5° incidence with analyte refractive indices of 1, 1.5, and 2, respectively. (**d**–**f**) Far-field scattering patterns for *N* = 4 at normal incidence with analyte refractive indices of 1, 1.5, and 2, respectively. (**g**) Energy ratio between specular and anomalous reflection versus refractive index for thin analyte layers (*N* = 2, 4; R^2^ > 0.99). (**h**) Energy ratio between specular reflection and average split-beam energy versus refractive index for thick analyte layers (*N* = 4, 6; R^2^ > 0.99). (**i**) Far-field scattering patterns showing suppression of the anomalous beam for thick analyte layers that are above 10 μm.

Figure 5g presents the quadratic fitting curves of this ratio as a function of the analyte refractive index, showing that *N* = 2 exhibits both a larger coverage range and a higher sensitivity (*S* = 8.25) than *N* = 4 (*S* = 4.69).

For thick analyte layers, the phase perturbation becomes significantly stronger, and the anomalous reflection beam is strongly suppressed or even annihilated at high refractive indices (Figure 5i). In this regime, beam splitting is employed for refractive index sensing instead. Taking *N* = 4 and *N* = 6 as examples, symmetric dual beams are generated under normal incidence, and the ratio between the specular reflection energy and the average energy (|*E_av_*|^2^) of the dual reflected beams is defined as the sensing indicator. Figure 5h shows that *N* = 4 exhibits both a larger coverage range and a higher sensitivity (*S* = 16.99) than *N* = 6 (*S* = 10.00).

In practical measurements, the incident angle can be flexibly adjusted. An oblique incidence configuration is preferred in the experiment because it facilitates the identification of the deflected beam. If the target diffracted beam cannot be clearly observed owing to limited diffraction efficiency, normal incidence can be adopted as an alternative measurement condition, and the beam-splitting mode can be used for refractive-index sensing.

In the present numerical study, the sensing response exhibits a clear and monotonic variation with increasing analyte refractive index, indicating that small refractive index perturbations can be distinguished by monitoring the far-field response. Since the current analysis is based on ideal electromagnetic simulations without considering experimental noise, detector limitations, or fabrication-induced fluctuations, an absolute experimental resolution or detection limit cannot be directly determined at this stage.

The bias-enabled refractive index sensing and the zero-bias fingerprint spectrum sensing are realized through the same physical aperture, achieving a unified integration of near-field and far-field, passive and active sensing.

## 3. Methods

### 3.1. Electromagnetic Simulation Method

The electromagnetic simulations were performed using the frequency-domain and time-domain solvers in CST Studio Suite 2020. For periodic metasurface simulations, the frequency-domain solver was employed with unit cell boundary conditions applied along the x and y directions and open (add space) boundary conditions applied along the z direction. A TM-polarized plane wave was excited from the top port, with a frequency range of 0.8–1.2 THz and a frequency step of 0.0004 THz.

For far-field beam manipulation simulations, the time-domain solver was used with open (add space) boundary conditions in all directions. A TM-polarized plane wave was incident from the top, with incident angles set according to the simulation requirements (0°, 20°, 25°, and ±27.5°). Far-field and electric-field monitors were employed to extract the radiation characteristics and electromagnetic field distributions.

The electromagnetic simulations were performed using the automatic mesh generation algorithm in CST Studio Suite 2020. The mesh density was automatically optimized according to the structural features and electromagnetic field distribution to ensure the reliability of the simulation results.

### 3.2. Materials

The metal used in this work was modeled as gold using the lossy metal model, with an electrical conductivity of $4.56\times 10^{7}$ S/m. The PI layer was modeled as a dielectric material with a relative permittivity of 3.5 and a loss tangent of 0.003.

The phase transition of VO_2_ between the insulating and metallic states was modeled by assigning different electrical conductivities corresponding to the two phases. In the insulating state, the conductivity was set to 200 S/m, corresponding to the experimentally measured value of the monoclinic phase. In the metallic state, the conductivity was set to 200,000 S/m, corresponding to the rutile phase. By tuning the conductivity of VO_2_, the reflection amplitude and phase responses were controlled, enabling the construction of the 1-bit-coding elements.

It should be noted that this work mainly focuses on the electromagnetic response under different VO_2_ conductivity states. The practical electrothermal switching process requires further optimization using coupled thermal–electrical simulations.

### 3.3. Analyte Layer Modeling

The L-tyrosine layer and refractive index layer were modeled as a homogeneous thin film, with its dielectric function described by parameters extracted from the experimentally measured complex permittivity reported. As shown in Figure 2h, the analyte thickness was set to 0.2, 1, 5, 10, and 15 μm to investigate the electromagnetic response of finite-thickness molecular layers. For refractive-index sensing, the refractive index of the covering layer was varied to evaluate the response of the metasurface to changes in the surrounding dielectric environment.

### 3.4. TCMT Parameter Extraction

The radiative and non-radiative loss rates were extracted by fitting the simulated reflection spectra with the single-port temporal coupled-mode theory model. The resonance frequency, $\gamma_{r}$ and $\gamma_{nr}$ were used as fitting parameters. The fitting was performed by minimizing the difference between the simulated and theoretical reflection spectra. To extract the parameters, the reflection coefficient could be expressed as:

(7) $$r(\omega )=\;\frac{i(\omega -\omega_{0})+\gamma_{nr}\;-\gamma_{r}}{\;i(\omega -\omega_{0})+\gamma_{nr}+\gamma_{r}}$$

The parameters have been defined above. The total quality factor *Q* of the resonant mode was first extracted from the simulated reflection spectrum according to the following:

(8) $$Q=\frac{\omega_{0}}{\Gamma_{FWHM}}$$

where *ω*_0_ represents the resonance angular frequency and $\Gamma_{FWHM}$ denotes the full width at half maximum (FWHM) of the resonance linewidth.

For a single-port resonant system and over-coupled regime, the total loss consists of radiative and non-radiative contributions. The corresponding quality factors can be separated as follows:

(9) $$Q_{r}=\frac{2Q}{1+\sqrt{R_{0}}}$$

(10) $$Q_{nr}=\frac{2Q}{1-\sqrt{R_{0}}}$$

where $R_{0}$ is the minimum reflection intensity at resonance, $Q_{r}$ and $Q_{nr}$ represent the radiative and non-radiative quality factors, respectively. The decay rates associated with the radiative and non-radiative channels were further calculated from the extracted quality factors:

(11) $$\gamma_{r}=\frac{\omega_{0}}{2Q_{r}}$$

(12) $$\gamma_{nr}=\frac{\omega_{0}}{2Q_{nr}}$$

The coupling regime of the metasurface can therefore be determined by comparing the radiative and non-radiative loss channels. Specifically, the system approaches critical coupling when $\gamma_{r}=\gamma_{nr}$, while the over-coupled regime corresponds to $\gamma_{r}>\gamma_{nr}$.

## 4. Conclusions

We propose and analyze an electrically tunable VO_2_-based terahertz metasurface that integrates broadband molecular fingerprint sensing, programmable wavefront manipulation, and far-field refractive index sensing within a single shared aperture. The device adopts a metal–insulator–metal architecture with a bowtie antenna and a VO_2_ microbridge, designed under temporal coupled mode theory.

In the unbiased state, the metasurface operates in an over-coupled regime, supporting a broadband low-Q resonance over 0.8–1.2 THz with strong local field enhancement. This enables near-field passive molecular fingerprint sensing, including the identification of the L-tyrosine absorption peak at 0.96 THz, with a maximum field intensity enhanced by about 2.19 times compared with a critically coupled configuration. Under electrical bias, Joule heating triggers the insulator-to-metal transition in VO_2_, producing a nearly 180° reflection phase shift with nearly invariant amplitude. Each unit cell thus acts as a 1-bit programmable meta-atom. By reconfiguring the coding sequence, the device realizes anomalous reflection and beam splitting, which are further exploited for far-field refractive index sensing. For thin analyte layers, the energy ratio between specular and anomalous reflection serves as the sensing indicator; for thick layers, the anomalous beam is suppressed and the ratio between specular reflection and the average split-beam energy is used instead.

Both detection modalities originate from the same over-coupled radiative loss mechanism: the enhanced near fields enable fingerprint sensing, while the open radiative channel supports far-field refractive index sensing through programmed phase coding. This shared-aperture design thus achieves a unified integration of near-field and far-field, passive and active detection. These results establish a promising platform for electrically tunable shared-aperture THz metasurfaces that unify near-field passive and far-field active detection, with potential applications in broadband molecular fingerprint sensing, refractive index sensing, programmable wavefront manipulation, and integrated sensing-communication systems.

## Figures and Tables

**Table 1:** Radiative and non-radiative loss rates extracted from TCMT fitting of the simulated reflection spectra.

PI Thickness	$\gamma_{r}$ (THz)	$\gamma_{nr}$ (THz)	$\gamma_{r}/\gamma_{nr}$	Coupling Regime
4.5 μm	0.0099	0.0097	1.02	Critical Coupled
6 μm	0.01730	0.0103	1.68	Over Coupled
8 μm	0.02587	0.0098	2.64	Over Coupled
10 μm	0.03321	0.0092	3.61	Over Coupled
12 μm	0.04454	0.0096	4.64	Over Coupled
15 μm	0.06026	0.0095	6.34	Over Coupled

**Table 2:** Summary of wavefront manipulation functions under different coding sequences.

Function	*N*	Coding Sequence	Incident Angle	Reflection Angle	Efficiency
Retroreflection	2	01	28°	28°	88.12%
Retroreflection	2	01	−28°	−28°	91.23%
Beam splitting	4	0011	0°	±26°	91.06%
Beam splitting	6	000111	0°	±17°	83.07%
Anomalous reflection	4	0011	20°	5°	53.05%
Anomalous reflection	6	000111	25°	−8°	46.85%

## Data Availability

The original contributions presented in this study are included in the article/Supplementary Material. Further inquiries can be directed to the corresponding authors.

## Supplementary Materials

The following supporting information can be downloaded at https://www.mdpi.com/article/10.3390/nano16181170/s1, Figure S1: The TCMT fitting was performed using data extracted from the simulations; Figure S2: Simulated far-field scattering patterns under different analyte refractive indices; Figure S3: Fingerprint detection in the over-coupled mode under multiple absorption peaks; Figure S4: Comparison of the metasurface performance at room temperature and 60 °C.

## Abbreviations

The following abbreviations are used in this manuscript:

LC	Inductor-Capacitor
MIM	Metal–Insulator–Metal
PI	Polyimide
Q	Quality Factor
TCMT	Temporal Coupled Mode Theory
THz	Terahertz
VO_2_	Vanadium Dioxide
TE	Transverse Electric
TM	Transverse Magnetic

## References

[1] Zeng Y., Sha X., Zhang C., Zhang Y., Deng H., Lu H., Qu G., Xiao S., Yu S., Kivshar Y. (2025). Metalasers with Arbitrarily Shaped Wavefronts. Nature.

[2] Abdollahramezani S., Hemmatyar O., Taghinejad M., Taghinejad H., Krasnok A., Eftekhar A.A., Teichrib C., Deshmukh S., El-Sayed M.A., Pop E. (2022). Electrically Driven Reprogrammable Phase-Change Metasurface Reaching 80% Efficiency. Nat. Commun..

[3] Qian M., Huang J., Chen X., Yuan X.-R., Zheng S., Yu K., Liu X.Q., Chen Q.D., Li Q., Liu Y. (2026). Synergetic Near field Reconstruction and Far field Control for Ultrabroad Scattering in Multiband Camouflage Based on Concave Reflection Metasurface. Adv. Photonics.

[4] Zhang X., Tian Z., Yue W., Gu J., Zhang S., Han J., Zhang W. (2013). Broadband Terahertz Wave Deflection Based on C-Shape Complex Metamaterials with Phase Discontinuities. Adv. Mater..

[5] Zhang Z., Wang Z., Zhang C., Yao Z., Zhang S., Wang R., Tian Z., Han J., Chang C., Lou J. (2024). Advanced Terahertz Refractive Sensing and Fingerprint Recognition through Metasurface-Excited Surface Waves. Adv. Mater..

[6] Zhang J., Lou J., Wang Z., Liang J., Zhao X., Huang Y., Chang C., Hu G. (2024). Photon-Induced Ultrafast Multitemporal Programming of Terahertz Metadevices. Adv. Mater..

[7] Zhang C., Lou J., Zhang J., Wang Z., Ji C.-Y., Yuan H., Huang Y., Li Z., Zhu W., Zhao S. (2026). Space-Time Wavefront Synchronized Terahertz Metasurface. Adv. Mater..

[8] Zheng Z., Sanderson G., Sotoodeh S., Clifton C., Ying C., Rahmani M., Xu L. (2025). Real-Time Programmable Nonlinear Wavefront Shaping with Si Metasurface Driven by Genetic Algorithm. Engineering.

[9] Zhuang X., Zhang W., Wang K., Gu Y., An Y., Zhang X., Gu J., Luo D., Han J., Zhang W. (2023). Active Terahertz Beam Steering Based on Mechanical Deformation of Liquid Crystal Elastomer Metasurface. Light Sci. Appl..

[10] Zhao X., Lou J., Xu X., Yu Y., Wang G., Qi J., Zeng L., He J., Liang J., Huang Y. (2022). Multifield Controlled Terahertz Modulator Based on Silicon-Vanadium Dioxide Hybrid Metasurface. Adv. Opt. Mater..

[11] Altug H., Oh S.H., Maier S.A., Homola J. (2022). Advances and Applications of Nanophotonic Biosensors. Nat. Nanotechnol..

[12] Baù E., Aigner A., Biechteler J., Heimg C., Weber T., Golz T., Maier S.A., Tittl A. (2025). Spatially Encoded Polaritonic Ultra-Strong Coupling in Gradient Metasurfaces with Epsilon-Near-Zero Modes. Adv. Mater..

[13] Cai T., Zheng B., Lou J., Shen L., Yang Y., Tang S., Li E., Qian C., Chen H. (2022). Experimental Realization of a Superdispersion-Enabled Ultrabroadband Terahertz Cloak. Adv. Mater..

[14] Cheng J., Dong X., Chen S., Yuan Y., Wen Q., Chang S. (2022). Terahertz Metagrating Accordion for Dynamic Beam Steering. Adv. Opt. Mater..

[15] Li D., Zhou H., Chen Z., Ren Z., Xu C., He X., Liu T., Chen X., Huang H., Lee C. (2023). Ultrasensitive Molecular Fingerprint Retrieval Using Strongly Detuned Overcoupled Plasmonic Nanoantennas. Adv. Mater..

[16] Li D., Wu X., Chen Z., Liu T., Mu X. (2025). Surface-Enhanced Spectroscopy Technology Based on Metamaterials. Microsyst. Nanoeng..

[17] Li D., Zhou H., Ren Z., Xu C., Lee C. (2025). Tailoring Light-Matter Interactions in Overcoupled Resonator for Biomolecule Recognition and Detection. Nano-Micro Lett..

[18] Liang X., Zhou Z., Li Z., Li J., Peng C., Zheng G. (2023). All-Optical Multiplexed Meta-Differentiator for Tri-Mode Surface Morphology Observation. Adv. Mater..

[19] Liu X., Xie Y., Yan Y., Niu Q., Zhu L.G., Dong Z., Liu Q.H., Zhu J. (2024). Rapid On-Demand Design of Inverted All-Dielectric Metagratings for Trace Terahertz Molecular Fingerprint Sensing by Deep Learning. ACS Photonics.

[20] Lou J., Xu X., Huang Y., Yu Y., Wang J., Fang G., Liang J., Fan C., Chang C. (2021). Optically Controlled Ultrafast Terahertz Metadevices with Ultralow Pump Threshold. Small.

[21] Lyu J., Shen S., Chen L., Zhu Y., Zhuang S. (2023). Frequency Selective Fingerprint Sensor: The Terahertz Unity Platform for Broadband Chiral Enantiomers Multiplexed Signals and Narrowband Molecular AIT Enhancement. PhotonIX.

[22] Ma S., Fan Y., Chen L., Zhao C., Sun J., Wang F., Lei D., Chen S., Wang P., Zhu Y. (2025). Electrically Controlled Real-Time Terahertz “Microladder” Integrated with VO_2_ Patches for Broadband Holographic Encryption. Adv. Photonics.

[23] Park J., Kim S.J., Landreman P., Brongersma M.L. (2020). An Over-Coupled Phase-Change Metasurface for Efficient Reflection Phase Modulation. Adv. Opt. Mater..

[24] Li G., Li G., He Z., Wang X., Klar P.J., Ma H., Zhang Y. (2026). Pixel-Controlled Programmable Metasurface as Phase-Type Spatial Terahertz Modulator. Opto-Electron. Adv..

[25] Yuan H., Zhao X., Zhang J., Liang J., Peng W., Xing H., Xu G., Zhang C., Lou J., Cong L. (2026). 3-Bit Parallel Logic Operations for Ultrafast Computing Based on Light-Driven Terahertz Metadevice. Adv. Funct. Mater..

[26] Qu C., Ma S., Hao J., Qiu M., Li X., Xiao S., Zhou L. (2015). Tailor the Functionalities of Metasurfaces Based on a Complete Phase Diagram. Phys. Rev. Lett..

[27] Lv W., Qin H., Shi X., Shi F., Mu X., Zhou Z., Qin J., Li B., Qiu C.W., Song Q. (2026). Local-Nonlocal Assisted Multifunctional Photonic Crystals. Light Sci. Appl..

[28] Wang G., Li M., Liu Q., Zang J., Li O., Du X., Xu L., Tao M., Han C. (2025). Terahertz Integrated Sensing and Mobile Communications Empowered by a 220-GHz-Band Portable Device. Nat. Commun..

[29] Shen R., Ghasempour Y. (2026). Joint Communication and Sensing with Structured Beams Carrying Orbital Angular Momentum. Nat. Commun..

[30] Yang Z.-Z., Wang C., Zhao Y., Ruan G.J., Yangdong X.J., Yang Y., Pan C., Cheng B., Liang S.J., Miao F. (2026). Communication-Aware In-Memory Wireless Neural Networks. Nat. Electron..

[31] Sisler J., Thureja P., Grajower M.Y., Sokhoyan R., Huang I., Atwater H.A. (2024). Electrically Tunable Space-Time Metasurfaces at Optical Frequencies. Nat. Nanotechnol..

[32] Sun H., Cao Y., Chen Y., Li L., Ma L., Jin S., Gu G., Song X., Wu W., Feng Z. (2025). Enhanced Broadband Terahertz Trace Fingerprint Spectrum Utilizing Multiunit Fano Metasurfaces. ACS Photonics.

[33] Tian H.W., Zhang X.G., Jiang W.X., Li X., Liu Y.K., Qiu C.-W., Cui T.J. (2022). Programmable Controlling of Multiple Spatial Harmonics via a Nonlinearly Phased Grating Metasurface. Adv. Sci..

[34] Wang L., Lan F., Liang S., Bi C., Song T., Shen D., Yang M., Chen L., Cong X., Jin S. (2025). A Dual-Programmable Metasurface with Duplex Beam Steering for Full-Space Terahertz Communications. Laser Photonics Rev..

[35] Wang L., Gong S., Xia C., Liu D., Cong X., Zhu A., Zeng H., Lan F., Yang Z., Otsuji T. (2026). Subarray Programmable Terahertz Metasurface for Optical Logic and High-Order Amplitude Modulation. Light Sci. Appl..

[36] Yang R., Fan Y., Zhu W., Hu C., Chen S., Wei H., Chen W., Chan C.T., Zhao Q., Zhou J. (2023). Terahertz Silicon Metagratings: High-Efficiency Dispersive Beam Manipulation above Diffraction Cone. Laser Photonics Rev..

[37] Wu H., Shao R., Xu Z., Wu J.W., Tan S., Wang X., Qi Z., Cheng Q., Zheng Y., Luo Y. (2025). A Programmable Metasurface Antenna That Approaches the Wireless Information Mapping Limit. Nat. Electron..

[38] Xu H.-X., Hu G., Kong X., Shao Y., Genevet P., Qiu C.-W. (2023). Super-Reflector Enabled by Non-Interleaved Spin-Momentum-Multiplexed Metasurface. Light Sci. Appl..

[39] Chen B., Wang X., Li W., Li C., Wang Z., Guo H., Wu J., Fan K., Zhang C., He Y. (2022). Electrically Addressable Integrated Intelligent Terahertz Metasurface. Sci. Adv..

[40] Wang W.N., Li H.Q., Zhang Y., Zhang C.L. (2009). Correlations between Terahertz Spectra and Molecular Structures of 20 Standard α-Amino Acids. Acta Phys. Sin..

[41] Yao Y., Shankar R., Kats M.A., Song Y., Kong J., Loncar M., Capasso F. (2014). Electrically Tunable Metasurface Perfect Absorbers for Ultrathin Mid-Infrared Optical Modulators. Nano Lett..

[42] Paggi L., Fabas A., El Ouazzani H., Hugonin J.P., Fayard N., Bardou N., Dupuis C., Greffet J.J., Bouchon P. (2023). Over-Coupled Resonator for Broadband Surface Enhanced Infrared Absorption (SEIRA). Nat. Commun..

[43] Zhang Y., Han F., Xiao Y., Zhang Z., Yang J., Lei Y., Gao F., Chen H., Du C.-H. (2025). Missing Harmonic Dynamics in Generalized Snell’s Law: Revealing Full-Channel Characteristics of Gradient Metasurfaces. Light Sci. Appl..

[44] Aigner A., Tittl A., Wang J., Weber T., Kivshar Y., Maier S.A., Ren H. (2022). Plasmonic Bound States in the Continuum to Tailor Light-Matter Coupling. Sci. Adv..

[45] Li W., Wang Z., Song L., Liang J., Liao D., Liu F., Ji W., Cai T. (2026). Broadband Achromatic Illusion via a Transmission-Reflection-Integrated Metasurface. Opt. Lett..

[46] Yang Q., Yao Z., Xu L., Dou Y., Ba L., Huang F., Xu Q., Cong L., Gu J., Yang J. (2025). Ultrasensitive Terahertz Fingerprint Retrieval with Multiple-BIC-Enabled Meta-Sensors. Laser Photonics Rev..

