# Supplementary material for: Electrically Tunable VO2-Based Metasurface for Shared-Aperture Terahertz Molecular Fingerprint Sensing and Programmable Wavefront Manipulation
Source: Nanomaterials (Basel). 2026 Sep 16;16(18):1170. doi: 10.3390/nano16181170 (PMC13610004; doi:10.3390/nano16181170)
Supplement: Supplementary file 1 [file nanomaterials-16-01170-s001.zip › nanomaterials-4543437-supplementary.pdf]

Supporting Information

Electrically Tunable VO_2-Based Metasurface for Shared-Aperture Terahertz Molecular Fingerprint Sensing and Programmable Wavefront Manipulation

Jiahe Wang †, Wenlong Li †, Xilai Zhao †, Wenye Ji, Xiongyu Liang, Tong Cai, Zhenxu Wang * and Jiangang Liang *

Air and Missile Defense College, Air Force Engineering University, Xi'an 710051, China; wangjiahe2025@163.com (J.W.); wenll011204@163.com (W.L.); zxl511178738@126.com (X.Z.); jiwenyewave@163.com (W.J.); xiongyu_liang@alumni.hust.edu.cn (X.L.); caitong326@sina.cn (T.C.)

* Correspondence: wzx206@126.com (Z.W.); cat-liang1975@163.com (J.L.)

† These authors contributed equally to this work.

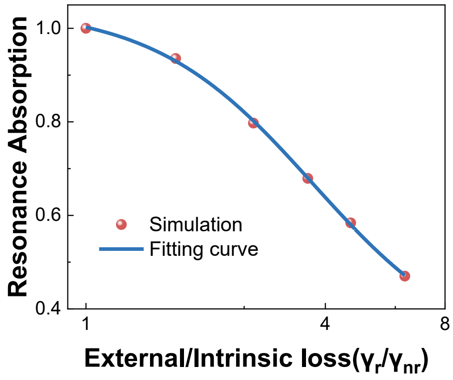

Figure S1 The TCMT fitting was performed using data extracted from the simulations, and the results yield a coefficient of determination $R^2 > 0.99$.

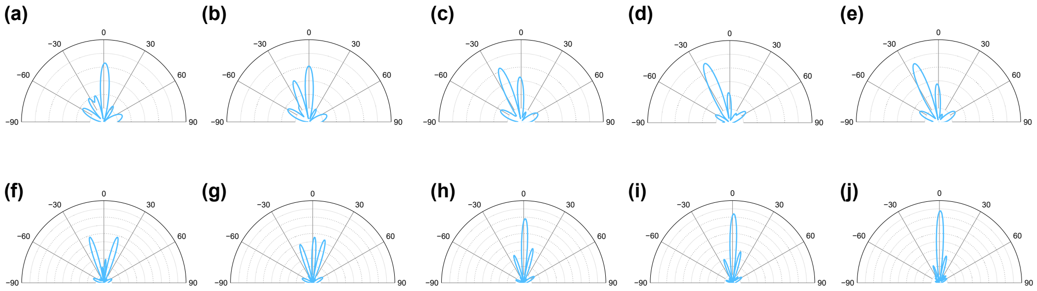

Figure S2. Simulated far-field scattering patterns under different analyte refractive indices. (a–e) Anomalous reflection mode with $N = 4$ at 20° incidence for $n = 1$, 1.2, 1.5, 1.7, and 2, respectively. (f–j) Beam splitting mode with $N = 6$ at normal incidence for n = 1, 1.2, 1.5, 1.7, and 2, respectively.

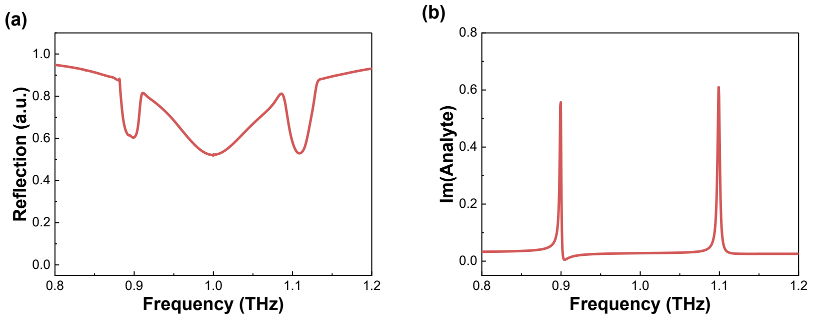

Figure S3. Fingerprint detection in the over-coupled mode under multiple absorption peaks.(a)Reflection spectra of the metasurface with multiple absorption peaks.(b) Imaginary part of the permittivity for multiple absorption peaks.

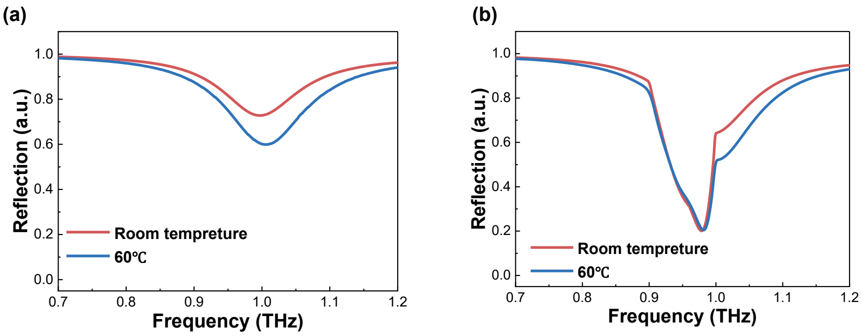

Figure S4. Comparison of the metasurface performance at room temperature and 60 °C. (a) Variation of the resonance peak. (b) L-tyrosine detection.
